# Association Between Long‐Term Glycemic Control and Clinical Outcomes in Patients With Diabetes Mellitus Hospitalized for Lower Respiratory Tract Infections: A Retrospective Cohort Study

**DOI:** 10.1155/jdr/6735488

**Published:** 2026-09-02

**Authors:** Alma Iselin, David Staehli, Fabienne Jaun, Eliska Potlukova, Joerg Daniel Leuppi, Maria Boesing, Giorgia Lüthi-Corridori

**Affiliations:** ^1^ University Institute of Internal Medicine, Cantonal Hospital Baselland, Liestal, Switzerland, ksbl.ch; ^2^ Faculty of Medicine, University of Basel, Basel, Switzerland, unibas.ch; ^3^ University Center of Internal Medicine, Cantonal Hospital Baselland, Liestal, Switzerland, ksbl.ch; ^4^ Harvard T.H. Chan School of Public Health—ECPE, Boston, Massachusetts, USA

## Abstract

**Introduction:**

Diabetes mellitus (DM) increases susceptibility to lower respiratory tract infections (LRTIs) and is associated with higher rates of complications and mortality. Although poor glycemic control is known to increase infection risk, it is unclear whether long‐term glycemic status influences outcomes once an LRTI occurs. This study examined whether hemoglobin A1c (HbA1c) predicts clinical outcomes in adults with DM hospitalized for LRTI.

**Methods:**

This retrospective, single‐center observational study was conducted at Kantonsspital Baselland, Switzerland, and included adult patients with Type 1 and Type 2 DM hospitalized for LRTI between January 2023 and December 2024. Data were extracted from the hospital′s electronic medical records system. Long‐term glycemic control was assessed by HbA1c values. Outcomes included length of hospital stay (LOHS), readmission, and mortality at 30 and 180 days. Associations were evaluated using multivariable regression models adjusted for age, sex, and number of chronic comorbidities.

**Results:**

A total of 389 patients were included, with a median age of 79 years (IQR 72–84) and 41.1% female. Multimorbidity was highly prevalent, with 76.9% of patients having ≥ 5 chronic conditions. The median HbA1c was 7.2%. The most frequent LRTI admission diagnosis was pneumonia (53.5%), followed by COVID‐19 (25.2%) and COPD exacerbation (17.0%). The median LOHS was 8 days (IQR 5–12). Mortality was 5.7% in hospital, 7.5% at 30 days, and 18.2% at 180 days after admission. Rehospitalization occurred in 13.4% of patients within 30 days and 36.7% within 180 days after discharge. Higher HbA1c was modestly associated with longer LOHS (IRR 1.058, *p* = 0.004) but not with readmission or mortality at any time point.

**Conclusion:**

Patients with DM hospitalized for LRTI are typically elderly, multimorbid, and experience high readmission and mortality rates. Long‐term glycemic control showed an association with slightly prolonged hospital stay but no association with mortality or readmission. These findings indicate limited prognostic value of HbA1c in the acute LRTI setting and highlight the dominant role of age, sex, and multimorbidity. These findings are primarily applicable to frail, elderly patients with multimorbidity and should be interpreted cautiously in other populations. Prospective studies should investigate the impact of short‐term glycemic control and structured inpatient diabetes management on outcomes.

## 1. Background

Diabetes mellitus (DM) is a chronic metabolic disorder affecting an increasing proportion of the global population, with more than 800 million adults currently living with the condition [1–4]. In Switzerland, 5% of the population has DM [5]. The prevalence is rising particularly among older, multimorbid individuals, those who are also at greatest risk of complications during acute illnesses.

Effective long‐term glycemic control, reflected by glycated hemoglobin (HbA1c), is central to the management of both Type 1 and Type 2 diabetes and is known to reduce the burden of micro‐ and macro‐vascular complications [6–8]. However, whether glycemic control also influences outcomes of acute nonmetabolic conditions in this vulnerable population remains less well understood.

Lower respiratory tract infections (LRTIs) are among the leading infectious causes of hospitalization and mortality worldwide. They include conditions such as pneumonia and infectious bronchitis, and can also trigger or occur in the context of acute exacerbations of chronic respiratory diseases [9–14]. In Switzerland, LRTIs cause tens of thousands of hospitalizations annually and over a thousand pneumonia‐related deaths each year [15], with clinical severity ranging from mild disease to cases requiring hospitalization, intensive care, or mechanical ventilation [16–25].

The interplay between DM and infections, particularly LRTIs, is complex and bidirectional [26]. Patients with DM are more susceptible to LRTI infections (notably pneumonia) due to hyperglycemia‐induced immune dysfunction, chronic inflammation, and higher multimorbidity, including chronic respiratory diseases [26, 27]. As a result, patients with diabetes hospitalized for LRTIs tend to experience more severe clinical course including longer hospital stays, higher complications rates, and increased mortality [23–27]. This risk is further amplified in those with suboptimal glycemic control [28–31]. Conversely, infections can worsen glucose regulation via stress‐mediated increases in insulin resistance and cytokine production [32].

Despite these well‐documented risks, disease specific recommendations for the inpatient management of individuals with DM experiencing LRTIs remain limited [18, 26, 33, 34]. Existing guidelines mainly focus on preventing hypoglycemia or ensuring glucose monitoring, but they provide little direction on whether long‐term glycemic status should influence risk stratification, prognosis, or management strategies during LRTIs [35–37].

A clear understanding of whether long‐term glycemic control influences clinical outcomes of LRTIs is needed, particularly in older patients with multiple comorbidities. In this specific group of patients, strict glycemic targets are usually relaxed due to concerns about hypoglycemia and limited long‐term benefit [38]. If poor glycemic control were associated with worse short‐term outcomes such as prolonged length of hospital stay (LOHS), higher readmission rates, or increased mortality, this could justify re‐evaluating glycemic targets in this population or identifying high‐risk patients who may benefit from intensified monitoring and resource allocation during hospitalization.

This study is therefore aimed at evaluating the association between long‐term glycemic control, measured by HbA1c, and clinical outcomes among patients with DM hospitalized for LRTI. Outcomes of interest include LOHS, readmissions, and mortality, assessed over a follow‐up period of 180 days.

## 2. Materials and Methods

### 2.1. Study Design and Setting

This retrospective, observational, single‐center study was conducted at the Cantonal Hospital of Baselland (Kantonsspital Baselland [KSBL]), a public teaching hospital serving approximately 300,000 people [39]. This manuscript was prepared in accordance with the STROBE (Strengthening the Reporting of Observational Studies in Epidemiology) guidelines, and the completed STROBE checklist is included in the Supporting Information.

### 2.2. Study Population

**Figure 1:**
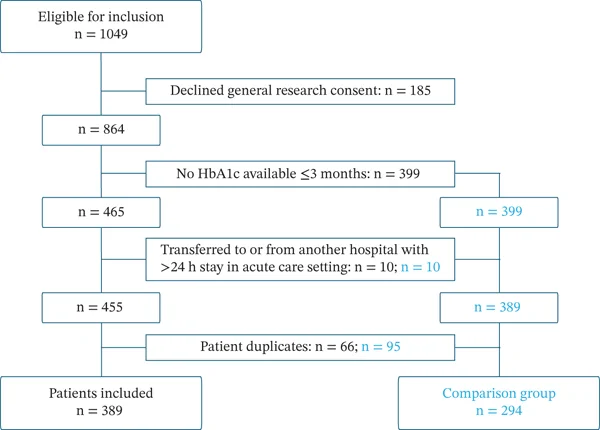
Flowchart included patients. Abbreviations: *n*, number; LRTI, lower respiratory tract infection; HbA1c, hemoglobin A1c (glycosylated hemoglobin).

The study population consisted of all consecutive adult patients with DM type 1 and 2 who were hospitalized for LRTI at the Cantonal Hospital Baselland (KSBL) between January 1, 2023, and December 31, 2024. Eligible patients were identified from the hospital′s administrative database based on ICD‐10 codes for DM (E10–E14) and LRTI diagnoses. Only the first eligible admission per patient was included. To evaluate the representativeness of the study cohort, a comparison group was also generated, comprising patients meeting all inclusion criteria except for the availability of HbA1c measurements. The detailed selection process of both cohorts is illustrated in Figure 1.

### 2.3. Inclusion and Exclusion Criteria

The following inclusion and exclusion criteria were applied to define the study population:

Inclusion criteria

• Age ≥ 18 years
• ICD‐10 codes for DM (E10–E14)
• ICD‐10 codes indicating LRTI
• Discharged from the KSBL between January 1, 2023, and December 31, 2024
• HbA1c documented within 3 months prior to or during the index hospitalization

Exclusion criteria

• Documented declined general research consent
• LRTI not contributing to the reason for admission
• Transfer to or from another acute care hospital with > 24‐h stay
• Duplicate patient entries

### 2.4. Data Collection

Data were extracted from hospital administrative records and electronic medical records, managed using REDCap—a web application designed for secure data collection and management. Manual screening ensured application of inclusion/exclusion criteria. A randomly selected subset of the data was subjected to a quality control review by another study physician.

### 2.5. Variables and Outcomes

Collected variables included demographics, clinical data at admission, diabetes‐specific parameters (e.g., HbA1c and glucose profiles), LRTI characteristics (e.g., pathogens and treatment), and outcomes. The outcomes of interest included LOHS, mortality (in‐hospital, 30‐/180‐day) and 30‐/180‐day readmission.

LOHS was defined as the total number of days of inpatient care during the index hospitalization.

Mortality within 30 and 180 days was defined as all‐cause death occurring within 30 or 180 days from the date of hospital admission, including in‐hospital deaths.

Rehospitalization within 180 days was defined as any unplanned hospital readmission occurring within 180 days after discharge from the index hospitalization, irrespective of subsequent survival status. Patients who were rehospitalized and subsequently died within 180 days remained classified as rehospitalized. Patients who died without being rehospitalized during follow‐up were excluded from the analysis for this outcome, as death precluded the possibility of rehospitalization.

### 2.6. Statistical Analysis

Descriptive statistics included medians with interquartile ranges (IQRs) for continuous variables and absolute and relative frequencies for categorical variables. HbA1c was described both as a continuous variable (median and IQR) and as a categorical variable using clinically relevant cutoffs (< 7%, 7%–8%, and > 8%). For regression analyses, HbA1c was modeled as a continuous variable to estimate the effect per 1% increase in HbA1c. Between‐group comparisons were performed using the chi‐square, *t*‐test, or Wilcoxon rank‐sum test, as appropriate. Associations between HbA1c and the clinical outcomes were assessed using multivariable regression models adjusted for sex, age, and number of comorbidities: zero‐truncated negative binomial regression to analyze LOHS and logistic regression for rehospitalization and mortality outcomes (30‐ and 180‐day).

Regression results were expressed as incidence rate ratios (IRRs) or odds ratios (ORs) with 95% confidence intervals (CIs). A two − tailed *p* value < 0.05 was considered statistically significant. All analyses were conducted using Stata Version 18.0 (StataCorp LLC, College Station, Texas, United States).

To assess potential selection bias related to the inclusion criterion of a recent HbA1c measurement, we conducted a comparison analysis between included patients and those excluded due to missing HbA1c values, examining demographic, clinical, and hospitalization characteristics. To evaluate whether the timing of HbA1c measurement influenced the observed associations, we performed a sensitivity analysis including the number of days between HbA1c assessment and hospital admission as an additional covariate in the multivariable model. The results of this analysis are reported in Table S2.

## 3. Results

### 3.1. Study Population

A total of 1049 cases eligible for inclusion were identified. After the application of exclusion criteria, a total of 389 patients were included in the study. The detailed selection process for the study population, as well as the comparison group, is illustrated in Figure 1.

The characteristics of the patients are summarized in Table 1. The study cohort was predominantly elderly, with a median age of 79 years (IQR 72–84), and 41.1% were female. The median BMI was 27.6 kg/m^2^. Almost all patients (98.7%) had at least one comorbidity in addition to DM, most commonly arterial hypertension (83.3%), cardiovascular disease (71.2%), and chronic kidney disease (55.9%). In addition, almost half of the patients had an underlying pulmonary disease (48.8%), with COPD being the most prevalent pulmonary comorbidity (55.3%). The proportion of highly multimorbid patients (≥ 5 chronic diseases including DM) [41] was 76.9%, with the median number of comorbidities of 6.

**Table 1:** Baseline demographic, clinical, and outcome characteristics of patients with diabetes mellitus hospitalized for lower respiratory tract infection (*n* = 389).

	Included (*n* = 389)	Missing (%)
Demographics		
Age (years), median (IQR), range	79 (72–84), 30–97	
Male, *n* (%)	229 (58.9%)	
Female, *n* (%)	160 (41.1%)	
BMI (kg/m^2^), median (IQR)	27.57 (24.0–31.8)	21 (5.4%)
Comorbidities		
Pulmonary, *n* (%)	190 (48.8%)	
COPD, *n* (%)	105 (55.3%)	
Obstructive sleep apnea syndrome, *n* (%)	70 (36.8%)	
Asthma, *n* (%)	30 (15.8%)	
Active pulmonary malignancy, *n* (%)	15 (7.9%)	
Other, *n* (%)	48 (25.3%)	
Cardiovascular diseases, *n* (%)	277 (71.2%)	
Ischemic heart disease, *n* (%)	164 (59.2%)	
Arrhythmias, *n* (%)	162 (58.5%)	
Cerebrovascular disease, *n* (%)	85 (30.7%)	
Heart failure, *n* (%)	67 (24.2%)	
Peripheral artery disease, *n* (%)	67 (24.2%)	
Arterial hypertension, *n* (%)	324 (83.3%)	
Dyslipidemia, *n* (%)	198 (50.9%)	
Obesity, *n* (%)	131 (33.7%)	
Chronic kidney disease, *n* (%)	217 (55.9%)	
Neurological, *n* (%)	134 (34.5%)	
Cognitive impairment, *n* (%)	111 (82.8%)	
Other, *n* (%)	35 (26.1%)	
Mental disorders, *n* (%)	83 (21.3%)	
Immunosuppression, *n* (%)	32 (8.2%)	
Multimorbidity		
Number of chronic comorbidities, median (IQR)	6 (5–8)	
Multimorbid (≥ 2 chronic conditions) [40]	384 (98.7%)	
Highly multimorbid (≥ 5 chronic conditions) [41]	299 (76.9%)	
Smoking status		180 (46.3%)
Never, *n* (%)	19 (9.1%)	
Former smoker, *n* (%)	107 (51.2%)	
Current smoker, *n* (%)	83 (39.7%)	
Outcomes		
LOHS (days), median (IQR)	8 (5–12)	
LOHS in survivors (days), median (IQR) (*n* = 366)	8 (5–12)	
In‐hospital death, *n* (%)	22 (5.7%)	
30‐day mortality from discharge, *n* (%)	35 (9%)	
180‐day mortality from discharge, *n* (%)	70 (18%)	
30‐day rehospitalization from discharge, *n* (%)	41/354^a^ (13.4%)	
180‐day rehospitalization from discharge, *n* (%)	98/319^b^ (36.7%)	

Abbreviations: BMI, body mass index; COPD, chronic obstructive pulmonary disease; IQR, interquartile range.

^a^ *N* = 35 excluded due to death within 30 days from discharge.

^b^ *N* = 70 excluded due to death within 180 days from discharge.

### 3.2. LRTI and Diabetes Characteristics

Detailed characteristics of LRTI, diabetes status, long‐term glycemic control, and diabetes‐related clinical parameters are presented in Table 2. The most frequent LRTI diagnosis was pneumonia (53.5%), followed by COVID‐19 (25.2%) and COPD exacerbation (17.0%). Antibiotic therapy was administered in 72.2% of patients, systemic steroids in 39.8%, antivirals in 7.7% and oxygen supplementation in 50.6%.

**Table 2:** Characteristics of lower respiratory tract infection and diabetes‐related clinical parameters in the study cohort (*n* = 389).

	Included (*n* = 389)	Missing (%)
LRTI diagnosis type		
Pneumonia, *n* (%)	208 (53.5%)	
Community‐acquired, *n* (%)	105 (50.5%)	
Hospital‐acquired, *n* (%)	7 (3.4%)	
Etiology unspecified, *n* (%)	84 (40.4%)	
Other etiology, *n* (%)	12 (5.8%)	
COVID‐19, *n* (%)	98 (25.2%)	
Influenza, *n* (%)	41 (10.5%)	
COPD exacerbation, *n* (%)	66 (17.0%)	
Asthma exacerbation, *n* (%)	12 (3.1%)	
Other, *n* (%)	35 (9.0%)	
DM characteristics		
Type		2 (0.6%)
Type 2, *n* (%)	379 (97.9%)	
Type 1, *n* (%)	7 (1.8%)	
Duration of diabetes		82 (21.1%)
Diagnosed during index admission, *n* (%)	28/307 (9.0%)	
< 5 years	47/307 (15.3%)	
5–10 years, *n* (%)	35/307 (11.4%)	
≥ 10 years, *n* (%)	197/307 (64.3%)	
Complications		
Diabetic neuropathy, *n* (%)	73 (18.8%)	
Diabetic retinopathy, *n* (%)	28 (7.2%)	
Diabetic foot disease, *n* (%)	8 (2.1%)	
None documented, including no CKD, *n* (%)	147 (37.8%)	
Long‐term glycemic control		
HbA1c (%), median (IQR), range	7.2 (6.5–8.1), 4.6–16.9	
HbA1c < 7*%* ^a^, *n* (%)	165 (42.4%)	
HbA1c 7%–8%^b^, *n* (%)	119 (30.6%)	
HbA1c > 8*%*, *n* (%)	105 (27.0%)	
HbA1c measured by general practitioner, *n* (%)	61 (15.7%)	
HbA1c measured during index case, *n* (%)	271 (69.7%)	
HbA1c measured by other GP or during prior hospitalization	57 (14.6%)	

*Note:* Other LRTI diagnoses include bronchitis/bronchiolitis, tuberculosis with pulmonary involvement, pertussis, psittacosis, lung abscess, pleural effusion associated with infection, and acute respiratory distress syndrome (ARDS).

Abbreviations: CKD, chronic kidney disease; COPD, chronic obstructive pulmonary disease; DM, diabetes mellitus; HbA1c, hemoglobin A1C (glycosylated hemoglobin); LRTI, lower respiratory tract infection.

^a^Goal set by ADA for most adults [42].

^b^Goal set by ADA for older adults with intermediate or complex health [38].

Type 2 DM was the predominant form, representing 97.9% of cases, whereas Type 1 DM was rare, observed in only 1.8% of patients. Diabetes duration was available for 307 (78.9%) patients, of whom 64.3% had been diagnosed ≥ 10 years before the index admission and 9.0% during the index admission. A total of 62.2% of patients presented with at least one diabetes‐related complication (diabetic neuropathy, diabetic retinopathy, or diabetic foot disease). The median HbA1c at admission was 7.2% (IQR 6.5–8.1). Values were < 7% in 42.4%, 7%–8% in 30.6%, and > 8% in 27.0% of patients. HbA1c was measured during hospitalization in 69.7% and by general practitioners within 3 months before admission in 15.7% of cases. Median admission glucose was 10.2 mmol/L, and the proportion of daily glucose readings within the target range (3.9–10 mmol/L) improved from 50% at admission to 66.7% at discharge. Dysglycemia events were reported in 32.4% of discharge summaries (hyperglycemia 87.3%, hypoglycemia 15.1%).

### 3.3. Outcomes Length of Stay, Mortality, and Rehospitalization

An overview of the outcomes is provided in Table 1. The median LOHS was 8 days (IQR 5–12). The in‐hospital mortality was 5.7%, 30‐day mortality 9%, and 180‐day mortality 18.2%. The 30‐day rehospitalization rate was 13.4%, and 36.7% of patients were readmitted within 180 days.

#### 3.3.1. LOHS

**Figure 2:**
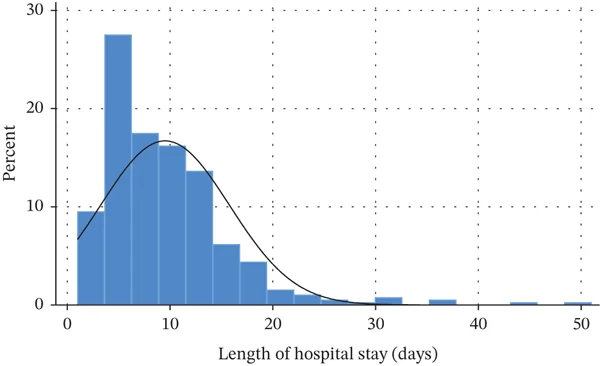
Histogram length of hospital stay.

The distribution of LOHS is shown in Figure 2.

In the zero‐truncated negative binomial regression adjusted for age, sex, and number of comorbidities, higher HbA1c was significantly associated with longer hospital stay (1.058, 95% CI: 1.018–1.100, *p* = 0.004). Older age (IRR 1.015, 95% CI: 1.009–1.021, *p* < 0.001) and higher number of comorbidities (IRR 1.029, 95% CI: 1.004–1.055, *p* = 0.022) were also independently associated with prolonged LOHS. Sex showed no significant association (IRR 0.983, 95% CI: 0.871–1.108, *p* = 0.777). Results are summarized in Table 3.

**Table 3:** Multivariable truncated negative binomial and logistic regression models assessing the association of HbA1c and covariates with clinical outcomes.

Variables	Effect measure	95% confidence interval	*p*value
Association with LOHS	IRR		
HbA1c	1.058	1.018–1.100	**0.004**
Sex, male	0.983	0.871–1.108	0.777
Age	1.015	1.009–1.021	**< 0.001**
Number of comorbidities	1.029	1.004–1.055	**0.022**
Association with in‐hospital mortality	OR		
HbA1c	0.962	0.677–1.369	0.832
Sex, male	2.485	0.917–6.740	0.074
Age	1.106	1.039–1.177	**0.002**
Number of comorbidities	1.063	0.887–1.275	0.507
Association with 30‐day mortality from discharge	OR		
HbA1c	0.884	0.656–1.192	0.420
Sex, male	1.775	0.821–3.835	0.144
Age	1.107	1.052–1.165	**< 0.001**
Number of comorbidities	1.098	0.946–1.274	0.217
Association with 180‐day mortality from discharge	OR		
HbA1c	1.022	0.832–1.257	0.834
Sex, male	2.017	1.124–3.620	**0.019**
Age	1.088	1.050–1.127	**< 0.001**
Number of comorbidities	1.128	1.009–1.260	**0.034**
Association with 30‐day rehospitalization from discharge	OR		
HbA1c	0.990	0.789–1.243	0.934
Sex, male	1.089	0.553–2.144	0.806
Age	0.996	0.964–1.028	0.799
Number of comorbidities	1.153	1.010–1.317	**0.035**
Association with 180‐day rehospitalization from discharge	OR		
HbA1c	0.910	0.760–1.089	0.304
Sex, male	0.741	0.451–1.217	0.236
Age	1.006	1.089–1.337	0.633
Number of comorbidities	1.177	1.076–1.288	**< 0.001**

*Note: p* values in bold are statistically significant.

Abbreviations: HbA1c, hemoglobin A1C (glycosylated hemoglobin); IRR, incidence rate ratio; OR, odds ratio.

The median time between HbA1c measurement and hospital admission was 3 days (IQR 1–10). In sensitivity analyses adjusting for the timing of HbA1c assessment, the association between HbA1c and LOHS remained robust, and timing itself was not independently associated with length of stay (Table S2).

#### 3.3.2. Mortality

The in‐hospital mortality rate was 5.7%, with 30‐ and 180‐day mortality after discharge of 9.0% and 18.0%, respectively. HbA1c was not significantly associated with mortality at any time point.

Age was significantly associated with all three mortality endpoints: in‐hospital: OR 1.106 (*p* = 0.002), 30‐day: OR 1.107 (*p* < 0.001) and 180‐day: OR 1.088 (*p* < 0.001). Male sex was associated with higher 180‐day mortality (OR 2.017, *p* = 0.019). The number of comorbidities was independently associated with 180‐day mortality (OR 1.128, *p* = 0.034). Table 3 presents detailed regression results.

#### 3.3.3. Readmission

Rehospitalization occurred in 13.4% of patients within 30 days and in 36.7% within 180 days after discharge. In adjusted logistic regression models HbA1c was not significantly associated with readmission at 30 days (OR 0.990, 95% CI: 0.789–1.243, *p* = 0.934) or 180 days (OR 0.910, 95% CI: 0.760–1.089, *p* = 0.304). The number of comorbidities was the only factor independently associated with increased readmission risk at both time points: 30 days (OR 1.153, *p* = 0.035) and 180 days (OR 1.177, *p* < 0.001). Age and sex showed no significant association with readmission.

## 4. Discussion

This study examined the association between long‐term glycemic control, as measured by HbA1c, and clinical outcomes in patients with diabetes hospitalized for LRTI at a Swiss teaching hospital. Our findings indicate that although higher HbA1c was associated with a slightly longer hospital stay, it showed no significant relationship with readmission or mortality. Instead, age, sex, and comorbidity burden emerged as the primary determinants of adverse outcomes.

These findings provide important insight into a clinically vulnerable but under‐researched population: elderly, multimorbid individuals in whom glycemic targets are commonly relaxed.

### 4.1. Study Population

The cohort reflects the demographic reality of patients with diabetes requiring hospitalization for respiratory infections: advanced age, high multimorbidity, and predominantly Type 2 diabetes. [43–46].

The study population was characterized by a remarkable prevalence of highly multimorbid patients, with 76.9% of patients having at least five chronic conditions. This high level of multimorbidity (median six comorbidities, 77% with ≥ 5) is consistent with existing literature on hospitalized patients with DM [47, 48] and LRTI [43–46], though the prevalence in this study was particularly high. Such a degree of multimorbidity likely contributes to the observed clinical complexity and elevated rates of readmission and mortality.

The distribution of LRTI types (pneumonia, COVID‐19, and COPD exacerbations) aligns with current epidemiological patterns [49–51], and reinforces the enduring relevance of respiratory infections among individuals with diabetes. [52–57].

### 4.2. Long‐Term Glycemic Control

The median HbA1c of 7.2% aligns with previously reported values in hospitalized patients with diabetes [47, 48]. Current ADA guidelines recommend less stringent targets (typically < 8%) for older adults with multimorbidity or limited life expectancy to avoid hypoglycemia and diminishing returns [38]. Given that our cohort had a median age of 79 years and over 75% of patients had at least five chronic conditions with only 27% exceeding an HbA1c of 8%, this distribution likely reflects appropriate individualized care rather than suboptimal control. Consequently, this restricted variation in HbA1c may partly explain its limited prognostic value for acute outcomes compared with age and overall comorbidity burden. Still, from a clinical perspective, our results suggest that HbA1c alone may not be a strong discriminator of risk during acute LRTI.

### 4.3. Outcomes

The median LOHS of 8 days is comparable with previous studies of patients with diabetes hospitalized with pneumonia or other respiratory infections in Switzerland and Europe [44, 58–62]. The association between higher HbA1c and longer LOHS, though statistically significant, was small and likely not clinically meaningful. A 1% increase in HbA1c was associated with approximately 0.5 additional hospital days. Although the per‐unit effect appears modest, differences of 2%–3% in HbA1c are common in clinical practice. Across this range, the cumulative effect may correspond to 1–1.5 additional hospital days, which could be relevant at the healthcare system level. However, when compared with other predictors, the magnitude of this association remains relatively limited. Age and comorbidity burden had stronger and more consistent associations of prolonged hospitalization, in line with prior studies on infection severity, healthcare resource use and recovery time among multimorbid patients [63–66].

The in‐hospital (5.7%), 30‐day (9%), and 180‐day (18.2%) mortality rates observed in this cohort were lower than those reported in international studies of patients with diabetes hospitalized with pneumonia, which documented in‐hospital mortality rates of 12%–15% and 180‐day mortality up to 24% [43, 45, 46]. This discrepancy may reflect differences in case mix, inclusion criteria, or care organization, as our study encompassed a broader range of LRTI diagnoses. However, our representativeness analysis revealed that patients with diabetes without a recent HbA1c measurement had a substantially higher in‐hospital mortality (11.6%), suggesting a potential selection bias.

Long‐term glycemic control was not associated with mortality at any time point, supporting prior evidence that HbA1c reflects chronic metabolic control rather than acute physiological reserve [67–69]. In contrast, older age and male sex were independent predictors of 30‐ and 180‐day mortality, consistent with previous findings in diabetic and LRTI populations [45, 70, 71]. The trend toward higher long‐term mortality with increasing comorbidity burden further highlights the dominant influence of multimorbidity on adverse outcomes in this vulnerable group.

The 30‐day readmission rate within our cohort was 13.4%, which is lower than the 17.9% reported in a comparable Danish study [43]. Since readmission data were obtained exclusively from the internal KSBL database, underreporting cannot be excluded. The 180‐day readmission rate was 36.7%, but no directly comparable studies were identified. A 2019 cohort of patients hospitalized for CAP in KSBL reported a lower overall 180‐day readmission rate of 31.6% and identified DM as an independent risk factor for readmission [72]. In that cohort, the 180‐day readmission rate among patients with diabetes was 37.3%. These findings, together with the substantial contribution of non‐LRTI and diabetes‐related factors to readmission in our cohort, underscore the complexity of this patient population and the role of DM as a complicating factor in LRTI.

HbA1c was not associated with readmission risk, confirming that long‐term glycemic control may have limited prognostic value for postdischarge outcomes in acutely ill patients. In contrast, the number of comorbidities was the only factor independently linked to both early and late readmission, emphasizing the cumulative effect of multimorbidity rather than glycemic status. Age and sex were not significantly associated with readmission, consistent with previous international evidence [43].

Overall, the lack of association between long‐term glycemic control and mortality or readmissions may partly reflect our cohort′s characteristics: Aligned with guidelines for older, multimorbid adults, only 27% of patients exceeded an HbA1c of 8%, suggesting that restricted baseline variation likely reduced the prognostic power of HbA1c relative to age and comorbidity burden.

### 4.4. Sensitivity Analysis: Representativeness of the Study Population

Although current guidelines recommend HbA1c measurement in all hospitalized patients with DM [35], the omission of HbA1c in a substantial proportion of cases may reflect pragmatic clinical decision‐making. In routine practice, laboratory tests are often prioritized based on perceived immediate clinical relevance, and HbA1c may be deferred in acutely ill or frail patients when it is not expected to influence acute management.

To assess potential selection bias related to the inclusion criterion of a recent HbA1c measurement, a sensitivity analysis was performed comparing included patients (with available HbA1c) and excluded patients (without HbA1c).

As summarized in Table S1 (see Supporting Information), patients with available HbA1c values had a statistically significant longer hospital stays but a lower in‐hospital mortality rate. These findings suggest that the study cohort represents a less critically ill subgroup of patients hospitalized with LRTI and DM. This potential bias should be considered when interpreting the present findings, which require confirmation in a more representative, ideally prospective, cohort.

### 4.5. Interpretation and Clinical Implications

Taken together, these results suggest that in elderly, multimorbid patients with DM hospitalized for LRTI, long‐term glycemic control plays a limited role in short‐ and mid‐term prognosis. Instead, demographic and comorbidity factors exert stronger effects on both hospital‐related and postdischarge outcomes.

From a clinical standpoint, the findings reinforce the importance of holistic management strategies in this population. Efforts should target the management of multimorbidity, comprehensive discharge planning, and posthospitalization follow‐up to reduce rehospitalization and mortality risks.

### 4.6. Limitations and Strengths

The retrospective single‐center design limits causal inference and may constrain generalizability beyond similar Swiss hospital settings. Second, the identification of rehospitalization relied on the KSBL internal electronic medical record system, which may underestimate readmission rates occurring outside the hospital network. Nevertheless, most unplanned readmissions in Switzerland occur at the same hospital, mitigating this concern to some extent [73].

A major limitation of this study is the potential selection bias introduced by restricting inclusion to patients with a documented recent HbA1c measurement. To evaluate this effect, a post hoc sensitivity (representativeness) analysis showed that patients with available HbA1c values had a significantly longer hospital stays but lower in‐hospital mortality, suggesting that patients with more advanced or palliative trajectories may have been underrepresented. As a result, the study cohort may not fully capture the full clinical spectrum of patients with diabetes hospitalized for LRTI. Systematic HbA1c assessment in future studies would help reduce this detection bias and strengthen generalizability of findings. In addition, hemoglobin concentration was not systematically available. As anemia may affect HbA1c values independently of glycemic status, we cannot exclude a potential influence on the interpretation of HbA1c in some patients. Treatment‐related factors such as antibiotic appropriateness, antimicrobial resistance, polypharmacy, or overall treatment complexity were not systematically captured in our dataset and could therefore not be incorporated into the regression models. Consequently, residual confounding related to therapeutic management cannot be excluded.

Despite these limitations, this study has notable strengths. It provides real‐world data from a large, clinically complex postpandemic cohort and encompasses multiple LRTI etiologies rather than focusing solely on pneumonia, enhancing representativeness of respiratory disease presentations in patients with diabetes. In addition, the analysis incorporated a broad range of clinically meaningful outcomes, including short‐ and medium‐term mortality, readmission, and length of stay. The inclusion of a sensitivity (representativeness) analysis strengthens internal validity by quantifying the impact of missing HbA1c measurements on cohort characteristics.

Overall, the study contributes valuable evidence to the limited European literature evaluating the prognostic role of long‐term glycemic control in diabetic patients with LRTI.

### 4.7. Future Research

Future research should address several gaps highlighted by our findings. Prospective cohort studies with systematic HbA1c testing and complete follow‐up across care networks would provide more representative and internally valid estimates of risk. Moreover, given that long‐term glycemic control showed limited prognostic value in the acute setting, future studies should focus on the potential role of short‐term glycemic control, dysglycemia during hospitalization, and structured inpatient glucose management strategies in influencing outcomes.

Importantly, our results underscore the clinical vulnerability of this patient group, characterized by high multimorbidity, frequent rehospitalization, and substantial medium‐term mortality. This aligns directly with the need for prospective interventional trials evaluating optimized inpatient care pathways for this specific population.

## 5. Conclusions

This study examined the association between long‐term glycemic control and clinical outcomes in patients with DM hospitalized for LRTI. HbA1c was associated with slightly longer hospital stays but not with readmission or mortality at 30 or 180 days. Age, male sex, and comorbidity burden were the primary determinants of adverse outcomes, underscoring the dominant influence of patient complexity over glycemic control in the acute setting.

Although modest at the individual level, clinically realistic differences in HbA1c may cumulatively translate into up to 1–1.5 additional hospital days, potentially relevant at the system level. In this predominantly elderly, multimorbid cohort hospitalized with LRTI, long‐term glycemic control appeared to play a limited prognostic role during acute LRTI episodes and support current recommendations to individualize—rather than universally tighten—glycemic targets in older multimorbid patients. To improve outcomes in this group, clinical strategies might address multimorbidity management, frailty assessment, and optimized transition‐of‐care. Prospective studies and clinical trials are needed to determine whether enhanced inpatient glucose management or targeted interventions can reduce the high burden of complications and rehospitalization observed in this vulnerable group. These findings primarily apply to elderly, multimorbid patients and should not be generalized to younger or less complex populations.

## Author Contributions

**Alma Iselin:** conceptualization, methodology, data curation, investigation, writing – original draft. **Maria Boesing:** conceptualization, methodology, validation, supervision, writing – review & editing. **Giorgia Lüthi-Corridori:** conceptualization, methodology, formal analysis, visualization, project administration, writing – original draft, writing – review & editing. **Eliska Potlukova:** validation, writing – review & editing. **Joerg Daniel Leuppi:** supervision, writing – review & editing. **David Staehli:** writing – review & editing. **Fabienne Jaun:** writing – review & editing. **Maria Boesing** and **Giorgia Lüthi-Corridori** contributed equally to this work and share last authorship.

## Funding

This study received no external funding. It was conducted as part of the routine academic research activities of the University Institute of Internal Medicine, Cantonal Hospital Baselland. Open access publishing was facilitated by the Universitat Basel, as part of the Wiley‐Universitat Basel agreement via the Consortium of Swiss Academic Libraries.

## Disclosure

All authors have read and approved the final version of the manuscript. Giorgia Lüthi‐Corridori (corresponding author) had full access to all of the data in this study and takes complete responsibility for the integrity of the data and the accuracy of the data analysis.

## Ethics Statement

This study has been approved by the Ethics Commission of Northwestern and Central Switzerland (EKNZ, Project‐ID 2024‐02303, Approval Date 21.11.2024). Patients who declined consent for the use of their routine clinical data for research purposes (general research consent) have been excluded.

## Conflicts of Interest

The authors declare no conflicts of interest.

## Supporting information


**Supporting Information** Additional supporting information can be found online in the Supporting Information section. Table S1 presents a comparison of baseline characteristics and outcomes between included patients with available HbA1c measurements and excluded patients without HbA1c data. Table S2 reports the sensitivity analysis adjusting for the timing of HbA1c measurement in relation to hospital admission. The completed STROBE checklist is provided as File S3.

## Data Availability

The data that support the findings of this study are available on request from the corresponding author. The data are not publicly available due to privacy or ethical restrictions.
