# Supplementary material for: Association Between Long‐Term Glycemic Control and Clinical Outcomes in Patients With Diabetes Mellitus Hospitalized for Lower Respiratory Tract Infections: A Retrospective Cohort Study
Source: J Diabetes Res. 2026 Sep 2;2026:6735488. doi: 10.1155/jdr/6735488 (PMC13536487; doi:10.1155/jdr/6735488)
Supplement: Supplementary file 1 [file JDR-2026-6735488-s001.zip › Suplementary Material_Table S2 (1).docx]

Table S2. Sensitivity analysis adjusting for timing of HbA1c measurement in the multivariable truncated negative binomial regression model for length of hospital stay

| Variables | Effect measure | 95% Confidence interval | p-value |
| --- | --- | --- | --- |
| Association with LOHS | IRR |  |  |
| HbA1c | 1.055 | 1.013–1.097 | **0.008** |
| Sex, male | 0.986 | 0.871–1.117 | 0.830 |
| Age | 1.015 | 1.009-1.021 | **<0.001** |
| Number of comorbidities | 1.028 | 1.002-1.054 | **0.032** |
| Days between HbA1c measurement and hospital admission | 0.998 | 0.899-1.001 | 0.376 |

Abbreviations: IRR = incidence rate ratio; HbA1c=hemoglobin A1C (glycosylated hemoglobin)
