# Supplementary material for: Association Between Long‐Term Glycemic Control and Clinical Outcomes in Patients With Diabetes Mellitus Hospitalized for Lower Respiratory Tract Infections: A Retrospective Cohort Study
Source: J Diabetes Res. 2026 Sep 2;2026:6735488. doi: 10.1155/jdr/6735488 (PMC13536487; doi:10.1155/jdr/6735488)
Supplement: Supplementary file 1 [file JDR-2026-6735488-s001.zip › Suplementary Material_Table S1 (1).docx]

Table S1. Comparison between included patients and the eligible comparison group (representativeness analysis)

|  | Comparison group  (n = 294) | Included  (n = 389) | P-value |
| --- | --- | --- | --- |
| Demographics |  |  |  |
| Age [years], median (IQR), range | 80 (72.25-86.75), 47-103 | 79 (72-84), 30-97 | 0.127 |
| Male, n (%) | 161 (54.8%) | 228 (58.8%) | 0.283 |
| Admission circumstances |  |  |  |
| Emergency presentation |  |  | **0.002*** |
| Ambulance, n (%) | 170 (57.8%) | 172 (44.2%) |  |
| Self-referral, n (%) | 78 (26.5%) | 119 (30.6%) |  |
| Ambulant referral, n (%) | 42 (14.3%) | 88 (22.6%) |  |
| Institutional referral, n (%) | 4 (1.4%) | 10 (2.6%) |  |
| Pre-admission location |  |  | **0.008*** |
| Home, n (%) | 264 (89.8%) | 355 (91.3%) |  |
| Nursing home, n (%) | 25 (8.5%) | 16 (4.1%) |  |
| Other, n (%) | 5 (1.7%) | 18 (4.6%) |  |
| Hospital course |  |  |  |
| Discharge circumstances |  |  | **<0.001*** |
| In hospital death, n (%) | 34 (11.6%) | 22 (5.7%) |  |
| Home, n (%) | 180 (61.2%) | 260 (66.8%) |  |
| Nursing home, n (%) | 53 (18.0%) | 32 (8.2%) |  |
| Rehabilitation facility, n (%) | 25 (8.5%) | 72 (18.5%) |  |
| Other, n (%) | 2 (0.7%) | 3 (0.8%) |  |
| Length of hospital stay [days], median (IQR) | 6 (4-10) | 8 (5-12) | **<0.001*** |

Abbreviations: IQR=Interquartile range
