# Supplementary material for: Association Between Long‐Term Glycemic Control and Clinical Outcomes in Patients With Diabetes Mellitus Hospitalized for Lower Respiratory Tract Infections: A Retrospective Cohort Study
Source: J Diabetes Res. 2026 Sep 2;2026:6735488. doi: 10.1155/jdr/6735488 (PMC13536487; doi:10.1155/jdr/6735488)
Supplement: Supplementary file 1 [file JDR-2026-6735488-s001.zip › Suplementary Material_S3_STROBE-checklist-v4-combined-PlosMedicine_2.docx]

S3.STROBE Statement—checklist of items that should be included in reports of observational studies

|  | Item No. | Recommendation | Page  No. | Relevant text from manuscript |
| --- | --- | --- | --- | --- |
| **Title and abstract** | 1 | (*a*) Indicate the study’s design with a commonly used term in the title or the abstract | 1 | *“A Retrospective Cohort Study”* |
|  |  | (*b*) Provide in the abstract an informative and balanced summary of what was done and what was found | 1 | Abstract describes background, methods (“retrospective… included adult patients… outcomes included…”), results (sample size, medians, mortality), and conclusion. |
| Introduction | | | |  |
| Background/  rationale | 2 | Explain the scientific background and rationale for the investigation being reported | 2 | Introduction, paragraphs 1–6 describe the scientific background and rationale:  *“Diabetes mellitus (DM)… increases susceptibility to LRTIs… whether glycemic control influences outcomes… remains less well understood… This study therefore aims to evaluate the association between long-term glycemic control…”* |
| Objectives | 3 | State specific objectives, including any prespecified hypotheses | 2 | *“This study therefore aims to evaluate the association between long-term glycemic control, measured by HbA1c, and clinical outcomes among patients with DM hospitalized for LRTI.”* |
| Methods | | | |  |
| Study design | 4 | Present key elements of study design early in the paper | 3 | *“This retrospective, observational, single-center study was conducted…” “This manuscript was prepared in accordance with the STROBE… guidelines.”* |
| Setting | 5 | Describe the setting, locations, and relevant dates, including periods of recruitment, exposure, follow-up, and data collection | 3-4 | *“…conducted at the Cantonal Hospital of Baselland… serving approximately 300,000 people… between 1 January 2023 and 31 December 2024.”* |
| Participants | 6 | (*a*) *Cohort study*—Give the eligibility criteria, and the sources and methods of selection of participants. Describe methods of follow-up  *Case-control study*—Give the eligibility criteria, and the sources and methods of case ascertainment and control selection. Give the rationale for the choice of cases and controls  *Cross-sectional study*—Give the eligibility criteria, and the sources and methods of selection of participants | 3-4 | *“The study population consisted of all consecutive adult patients… hospitalized for LRTI… identified from the hospital administrative database… Only the first eligible admission per patient was included.”*  Inclusion and exclusion criteria listed explicitly in section 2.3. |
|  |  | (*b*) *Cohort study*—For matched studies, give matching criteria and number of exposed and unexposed  *Case-control study*—For matched studies, give matching criteria and the number of controls per case | n.a. | Not applicable (no matching). |
| Variables | 7 | Clearly define all outcomes, exposures, predictors, potential confounders, and effect modifiers. Give diagnostic criteria, if applicable | 3-4 | Section 2.5 lists outcomes and all variables:  *“Collected variables included demographics, clinical data… diabetes-specific parameters… LRTI characteristics… outcomes included LOHS, mortality, readmission.”* |
| Data sources/ measurement | 8* | For each variable of interest, give sources of data and details of methods of assessment (measurement). Describe comparability of assessment methods if there is more than one group |  | *“Data were extracted from hospital administrative records and electronic medical records… managed using REDCap… A randomly selected subset… underwent quality control review.”* |
| Bias | 9 | Describe any efforts to address potential sources of bias | 4, 11 | Bias addressed in sensitivity analysis:  “…comparison analysis between included patients and those excluded due to missing HbA1c… examining representativeness.”  “…sensitivity (representativeness) analysis showed that patients with available HbA1c… differed…” |
| Study size | 10 | Explain how the study size was arrived at | 4-5 | *“A total of 1049 cases eligible… after exclusions, 389 patients were included.”*  Sample size determined by all eligible cases in the time frame. |
| Quantitative variables | 11 | Explain how quantitative variables were handled in the analyses. If applicable, describe which groupings were chosen and why | 4,7 | *“HbA1c values were analyzed as continuous variables… also categorized as <7%, 7–8%, >8%…”* (Table 2 categories). |
| Statistical methods | 12 | (*a*) Describe all statistical methods, including those used to control for confounding | 4 | Section “Statistical Analysis”:  *“Descriptive statistics… multivariable regression models adjusted for age, sex, and number of comorbidities… zero-truncated negative binomial regression… logistic regression…”* |
|  |  | (*b*) Describe any methods used to examine subgroups and interactions | n.a. | Not applicable. |
|  |  | (*c*) Explain how missing data were addressed | n.a. | Not applicable. |
|  |  | (*d*) *Cohort study*—If applicable, explain how loss to follow-up was addressed  *Case-control study*—If applicable, explain how matching of cases and controls was addressed  *Cross-sectional study*—If applicable, describe analytical methods taking account of sampling strategy | n.a. | Not applicable. |
|  |  | (*e*) Describe any sensitivity analyses | 4 | *Simulation-based post-hoc power analysis…”*  *“Comparison analysis between included patients and those excluded…”* (representativeness/sensitivity analysis). |
| Results | | | | |
| Participants | 13* | (a) Report numbers of individuals at each stage of study—eg numbers potentially eligible, examined for eligibility, confirmed eligible, included in the study, completing follow-up, and analysed | 5 | Section “Study Population”:  “*A total of 1049 cases… 389 included…”* and Figure 1 flowchart. |
|  |  | (b) Give reasons for non-participation at each stage | n.a. | Not applicable. |
|  |  | (c) Consider use of a flow diagram | 5 | Flow diagram provided (Figure 1). |
| Descriptive data | 14* | (a) Give characteristics of study participants (eg demographic, clinical, social) and information on exposures and potential confounders | 5-9 | Baseline characteristics in Table 1 and Table 2 |
|  |  | (b) Indicate number of participants with missing data for each variable of interest | 5-7 | Missing data clearly indicated in tables (Table 1, Table 2) |
|  |  | (c) *Cohort study*—Summarise follow-up time (eg, average and total amount) | 5-9 | 30 days and 180 days |
| Outcome data | 15* | *Cohort study*—Report numbers of outcome events or summary measures over time | 7-9 | Outcome numbers in Table 1 and in Results narrative (mortality, rehospitalization, LOHS). |
|  |  | *Case-control study—*Report numbers in each exposure category, or summary measures of exposure | n.a. | Not applicable. |
|  |  | *Cross-sectional study—*Report numbers of outcome events or summary measures | n.a. | Not applicable. |
| Main results | 16 | (*a*) Give unadjusted estimates and, if applicable, confounder-adjusted estimates and their precision (eg, 95% confidence interval). Make clear which confounders were adjusted for and why they were included | *7-9* | Regression results in Table 3 with adjusted estimates and 95% CI. |
|  |  | (*b*) Report category boundaries when continuous variables were categorized |  | “HbA1c was categorized into <7%, 7–8%, and >8% groups, consistent with ADA guideline target ranges for adults with diabetes.” (see Table 2) |
|  |  | (*c*) If relevant, consider translating estimates of relative risk into absolute risk for a meaningful time period |  | Not applicable: no absolute risk calculations were performed, as the study focused on OR and IRR estimates from multivariable regression analyses. |
| Other analyses | 17 | Report other analyses done—eg analyses of subgroups and interactions, and sensitivity analyses | 11-13 | Sensitivity analyses described in “Sensitivity analysis: Representativeness of the study population”. |
| Discussion | | | | |
| Key results | 18 | Summarise key results with reference to study objectives | 9 | “Our findings indicate that while higher HbA1c was associated with slightly longer hospital stay, it showed no significant relationship with readmission or mortality…” |
| Limitations | 19 | Discuss limitations of the study, taking into account sources of potential bias or imprecision. Discuss both direction and magnitude of any potential bias | 11-12 | Section “Limitations and Strengths” discusses bias, missing HbA1c, rehospitalization underreporting, retrospective design. |
| Interpretation | 20 | Give a cautious overall interpretation of results considering objectives, limitations, multiplicity of analyses, results from similar studies, and other relevant evidence | 11 | …findings suggest HbA1c alone provides limited prognostic value…” and integration with prior literature. |
| Generalisability | 21 | Discuss the generalisability (external validity) of the study results | 11-12 | *“The retrospective single-center design limits generalizability… cohort may represent a less critically ill subgroup…”* |
| Other information | |  | | |
| Funding | 22 | Give the source of funding and the role of the funders for the present study and, if applicable, for the original study on which the present article is based | 13 | This study received no external funding. It was conducted as part of the routine academic research activities of the University Institute of Internal Medicine, Cantonal Hospital Baselland.” |

*Give information separately for cases and controls in case-control studies and, if applicable, for exposed and unexposed groups in cohort and cross-sectional studies.

**Note:** An Explanation and Elaboration article discusses each checklist item and gives methodological background and published examples of transparent reporting. The STROBE checklist is best used in conjunction with this article (freely available on the Web sites of PLoS Medicine at http://www.plosmedicine.org/, Annals of Internal Medicine at http://www.annals.org/, and Epidemiology at http://www.epidem.com/). Information on the STROBE Initiative is available at www.strobe-statement.org.
